# Knowledge, perceptions and use of generic drugs: a cross sectional study

**DOI:** 10.1590/S1679-45082014AO3125

**Published:** 2014

**Authors:** Claudio Andre Barbosa de Lira, Jéssica Nathalia Soares Oliveira, Marília dos Santos Andrade, Cássia Regina Vancini-Campanharo, Rodrigo Luiz Vancini

**Affiliations:** 1 Human Physiology and Exercise Department, Faculdade de Educação Física e Dança, Universidade Federal de Goiás, Goiânia, GO, Brazil.; 2 Biomedicine course, Regional Jataí, Universidade Federal de Goiás, Jataí, GO, Brazil.; 3 Department of Physiology, Universidade Federal de São Paulo, São Paulo, SP, Brazil; 4 Escola Paulista de Enfermagem, Universidade Federal de São Paulo, São Paulo, SP, Brazil.; 5 Physical Education and Sports Center, Universidade Federal do Espírito Santo, Vitória, ES, Brazil.

**Keywords:** Reference drugs, Generic drugs, Public policies, Drug use, Patient education

## Abstract

**Objective:**

To assess the level of knowledge, perceptions and usage profile for generic drugs among laypersons.

**Methods:**

A cross-sectional study was conducted with 278 volunteers (180 women and 98 men, aged 37.1±15.8 years). A questionnaire was drawn up with questions on their use, perceptions and knowledge of generic drugs.

**Results:**

Most respondents (99.6%) knew that generic drugs exist, but only 48.6% were able to define them correctly, while 78.8% of the respondents had some information about generics. This information was obtained mainly through television (49.3%). In terms of generic drug characteristics, 79.1% stated that they were confident about their efficacy, 74.8% believed that generic drugs have the same effect as branded medications, 88.8% said that generics were priced lower than branded medications, and 80.2% stated that they bought generic drugs because of price. With regard to drugs prescribed by medical practitioners, 17.6% of the participants said that their doctors never prescribed generics and only 7.5% confirmed that their doctors always prescribed generics.

**Conclusion:**

For the lay public, the sample in this study has sufficient knowledge of generic drugs in terms of definition, efficacy and cost. Consequently, the volunteers interviewed are very likely to use generics. Furthermore, the results of this study indicate that programs should be implemented in order to boost generic drug prescriptions by medical practitioners.

## INTRODUCTION

Drugs play a role in health protection and recovery, in addition to helping maintain and enhance the quality of life.^(1)^ Around one third of the world’s population encounters difficulties in accessing medications, due to high prices, with this proportion rising to 50% in the developing countries.^(2)^ Consequently, generic drugs are an alternative to reference drugs in many countries all over the world, including the United States (USA), Germany, United Kingdom, Iraq, Malaysia and Brazil.^(3)-7)^

A generic drug is defined as a medication that is produced freely after expiry of the patent protecting the branded product, necessarily being similar to the reference drug in bioequivalence in order to obtain the same therapeutic effect.^(8)^ The reference drug is registered with the federal public health surveillance agency, and its quality must be proven scientifically when applying for registration, with its efficacy and safety being tested through clinical trials.^(9)^ In addition to reference and generic drugs, there is a third class called “similar drugs”, defined as medications with the same active ingredient(s), concentration, pharmaceutical form, route of administration, dosage and treatment indication, which are equivalent to the medication registered with the federal agency, although allowed to differ in some characteristics, such as product size and shape, use-by dates, packaging, labeling, excipients and vehicles.^(9)^

In practice, generic and similar drugs are copies of the reference drug, with the difference between them being the fact that generics use the name of the active ingredient, while similar drugs use the commercial name. Additionally, since they were first introduced, generic drugs have been bound to present bioequivalence tests, while this requirement was imposed for similar drugs in Brazil only from 2003 onwards. However, by year-end 2014, all Brazilian similar drugs must provide evidence of relative bioavailability.^(9)^

In Brazil, the introduction of generic drugs (Law No. 9,787 promulgated in 1999) resulted in a drop in prices between 40% and 62%.^(10)^ However, their sales in Brazil account for only 27.1% of the pharmaceutical market, although reaching around 80% in the USA.^(11)^

Despite the major advantages offered by generic drugs, resistance factors have already been described in terms of their use, such as: (1) limited availability of these products in chemists;^(10)^ (2) weak encouragement for their prescription by medical practitioners;^(6, 12)^ (3) lack of knowledge among healthcare professionals;^(13)^ (4) lack of usage guidelines;^(14)^ and (5) lack of knowledge and negative beliefs among consumers regarding their use. In a study conducted in Spain, Vallès et al.^(14)^ showed that 98.8% of patients agreed to switch from reference drugs to generics after receiving information on the generics. This shows that knowledge about generic drugs is a major factor guiding their choice by consumers.

In Brazil, outlays on healthcare account for the fourth largest group of family expenditures, and outlays on medications account for some 48.6% of these expenses.^(15)^ The introduction of generic drugs thus became an alternative for acquiring good quality medications at affordable prices for large segments of the population.

As a result, a cross-sectional study assessing knowledge of generic drugs, information received by consumers about these medications and consumer usage profiles can portray their situation in Brazil, fifteen years after the Generic Drugs Act was promulgated, as well as aspects still shadowed by doubts among consumers. Moreover, this research project can help steer planning for public health and health education policies on this important issue.

## OBJECTIVE

The objective of this study was to investigate knowledge, perceptions and use of generic drugs among laypersons.

## METHODS

### Subjects

This is a descriptive, cross-sectional study with 278 subjects (180 women) aged 37.1±15.8 years (range 18.1 - 89.7 years). The volunteers were recruited through direct contacts in public places such as streets and malls, in the following Brazilian municipalities: São Paulo State - São Paulo (n=124); Osasco (n=6); Guarulhos (n=5); Mairiporã (n=2); Carapicuíba (n=1); Diadema (n=1); Franco da Rocha (n=1); Itaquaquecetuba (n=1); Santos (n=1); Goiás State – Jataí (n=110); Rio Verde (n=2); Mineiros (n=1); Quirinópolis (n=1); Paraíba State – Patos (n=1); Rio de Janeiro State – Rio de Janeiro (n=1); Piauí State – Teresina (n=1), with 19 not identifying their municipality of origin. In terms of healthcare services, 158 (56.8%) of the respondents affirmed that they were covered by health insurance plans and were treated at private facilities; 105 (37.8%) were treated at government health units; 9 (3.2%) had no health insurance plan and were treated at public and private health units; and 6 (2.1%) stated that they had no health insurance plan and were not treated at any type of healthcare facility. Table 1 presents the additional characteristics of the sample.

**Table 1:** Characteristics of the sample (n=278)

Family income (minimum wage)	n (%)	Socioeconomic class*	n (%)	Schooling level	n (%)
Up to 1	10 (3.6)	E	0 (0.0)	Primary School – incomplete	22 (7.9)
1-2	35 (12.6)	D	6 (2.2)	Primary School – complete	10 (3.6)
2-3	43 (15.5)	C2	28 (10.1)	Secondary School – incomplete	24 (8.6)
3-4	63 (22.7)	C1	60 (21.6)	Secondary School – complete	88 (31.7)
4-5	51 (18.3)	B2	106 (38.1)	University – incomplete	69 (24.8)
5-10	49 (17.6)	B1	55 (19.8)	University – complete	35 (12.6)
10-15	9 (3.2)	A2	21 (7.6)	Graduate Studies – incomplete	5 (1.8)
15-20	18 (6.5)	A1	2 (0.7)	Graduate Studies – complete	25 (9.0)

* Using the criteria established by the Brazilian Association of Research Companies.^(16)^

Schoolchildren or students aged under 18 years and healthcare practitioners were excluded from the sample, as well as people with cognitive disorders that prevented them from understanding the data collection tools. The survey was conducted between March 2012 and October 2013. All the data collection and analysis tools were approved by the Research Ethics Committee at the *Universidade Federal de Goiás* (Protocol No. 168/2012) compliant with Resolution No. 466/2012 issued by the *Conselho Nacional de Saúde* [National Health Council]. After a detailed explanation of the objectives and procedures, the participants signed the Informed Consent Form.

### Assessment of knowledge about generic drugs

After an extensive literature review on this topic, a questionnaire (Annex 1) was drawn up to assess knowledge and perceptions of generic drugs. The questionnaire was divided into three parts: (1) personal data, (2) education and (3) knowledge about generic drugs, prior experience with them, means of acquisition, motives for use and reasons explaining their choice or not.

### Statistical analysis

The data were analyzed through the GraphPad Prism^®^ statistics software (Version 5.0, San Diego, USA). Descriptive statistics were used to analyze the findings (mean, standard deviation and absolute and relative frequencies).

## RESULTS

Among the respondents, 277 (99.6%) had already heard about generic drugs. Surprisingly, one volunteer stated that he/she had never heard about these medications. Additionally, 135 respondents (48.6%) correctly defined generic drugs and 86 (30.9%) did not know how to answer this question.

Nevertheless, 219 (78.8%) respondents had received information on generic drugs. This information was obtained through the following means: television (49.3%), medical practitioners (18%), pharmacies, from counter clerks or pharmacists (39.5%) the internet and newspaper articles (7.2%), acquaintances or neighbors (3.6%), radio (2.9%), street advertising (1.8%); other healthcare practitioners and at universities (0.7%). In terms of use, 225 (81%) stated that they were taking or had already taken generic drugs.

Regarding the characteristics of generic drugs, 220 (79.1%) stated that they were confident about their efficacy, 208 (74.8%) believed that they had the same effects as the reference drugs and 209 (75.2%) believed that generic drugs were just as safe as reference drugs. However, 40 (14.4%) thought that generic drugs were poorer quality than reference drugs and 95 (34.2%) would not select the generic instead of reference drug.

With regard to prescribing medications by physicians, 49 (17.6%) participants stated that their physicians never prescribed generic drugs and only 21 (7.5%) said that their physicians always prescribed generics. Despite low prescribing among medical practitioners, 48 volunteers (17.4%) stated that they habitually buy these medications, with 62 (22.3%) buying them frequently and 107 (38.5%) sometimes.

Among all the respondents, 247 (88.8%) stated that generic drugs are less expensive than reference drugs and 223 (80.2%) stated that they buy generic drugs because of the price.

When asked “If your physician prescribed a branded drug and the pharmacist offered you a cheaper generic, would you agree to switch?”, 183 (65.8%) replied that they would. When asked “If your physician prescribed a branded drug and the pharmacist offered you a generic drug for the same price, would you agree to switch?”, only 74 (26.6%) replied that they would. With regard to the statement “I would switch from a reference drug to a generic drug only if the disease was not serious”, 93 (33.5%) replied positively.

Among the respondents, 157 (56.5%) stated that generic drugs had the same substance as reference drugs and 140 (50.3%) felt that generic drugs were well publicized in Brazil. When asked whether they could find generic drugs in pharmacies, 120 (43.2%) stated that they always found them.

## DISCUSSION

In Brazil, outlays on medication account for 48.6% of expenditures on healthcare.^(15)^ As a result, the availability of medications at affordable prices must be included in the list of topics addressed by public health policies. Some studies assessed compliance with the use of generic drugs in Brazil^(16, 17)^ and, in the early days, due to a lack of knowledge among healthcare professionals and the lay population, there was some resistance to prescribing and taking them. In order to see whether the situation has changed, this study explored the perceptions, levels of knowledge and use of generic drugs fifteen years after the promulgation of Brazil’s Generic Drugs Act, showing that 99.6% of the respondents had already heard about them. This finding is similar to that obtained in other studies conducted in Brazil.^(11, 17)^ In a survey conducted in Recife, Pernambuco State, da Rocha et al.^(17)^ demonstrated that 95.7% of the respondents had heard about generic drugs. In a survey conducted by National Health Surveillance Agency (ANVISA - *Agência Nacional de Vigilância Sanitária*), 95% of consumers stated that they knew about generic drugs.^(11)^ Fortunately, the Brazilian context is better than that noted in other countries. In a study conducted in Auckland, New Zealand, only 51% of the respondents had heard of the phrase “generic drug”.^(3)^ Naing et al.^(18)^ assessed knowledge of the medications taken by the population of Malaysia, noting that 85.8% did not know the term “generic drug”, 86.3% did not know how to reply on the quality of generic drugs compared to reference drugs and 86.9% did not know about price differences between generic and reference drugs. The good results obtained among the Brazilian population can be attributed to awareness-heightening and popularization programs for generic drugs, run by the Brazilian Government and the pharmaceutical companies producing these types of medications.

In this study, 78.8% had obtained information on generic drugs: 49.3% from television and 18% from physicians. Findings similar to those presented in this study were described by Himmel et al., ^(19)^ in a study of the German population.

With regard to the definition of a generic drug, 48.6% of the respondents defined it correctly. In a study conducted in Malaysia of 216 people, Thomas and Vitry^(7)^ assessed knowledge about generic drugs, the wish to take them and the reasons for choice. Among the sample assessed, 32.5% stated that they knew what generic drugs were. However, when asked to provide a more exact definition, 7% (out of the 32.5%) were unable to do so. Among them who provided this information, 51% said that they were cheaper medications, 18% stated that they were “not original” or “not genuine”, 18% said that they were medications manufactured locally or by a company other than the mainstream manufacturer, and 13% said that their names were different from a medication with the same contents.

In this study, most (79.1%) of the respondents believed in efficacy of generics. Finding similar to those of our study were obtained by Thomas and Vitry.^(7)^ People declaring that they do not believe in the efficacy of generic drugs probably do so because of negative experience with these medications (lower efficacy, compared to reference drugs). However, this effect is not exclusive to generics. This may also be blamed on inter-individual variations, which is a serious problem consisting of a loss of pharmacological efficacy and adverse effects. Some of the factors associated with the variability of the pharmacological and therapeutic effects, are age, pregnancy and the presence of disease.^(20)^ In fact, there are situations in which a specific medication may present a therapeutic response other than the expected outcome or the patient may even be refractory to pharmacological treatment in some cases. For example, one third of patients with depression are refractory to pharmacological treatment.^(21)^ Although some are resistant to taking generics, in this study, 80.9% of the respondents had already followed this type of treatment. These findings cannot be attributed to their prescription by physicians as, according to the respondents, only 7.5% and 13.3% always and frequently prescribe generics, respectively. These findings are noteworthy as they suggest that the population does not follow medical prescriptions, replacing the reference drug by a cheaper generic counterpart. In this study, 88.8% stressed that the generic is cheaper than the reference drug, 80.2% buy the generic drug because of the price and 65.8% of the respondents replied positively when asked if they would choose the generic over the reference drug. Finally, when asked “If your physician prescribed a branded drug and the pharmacist offered you a cheaper generic, would you agree to switch?”, 65.8% replied that they would. This means that the price of generic drugs is a decisive factor in patient choices. Confirming this statement, when the respondents were asked “If your physician prescribed a branded drug and the pharmacist offered you a generic drug for the same price, would you agree to switch?”, 73.4% replied that they would not and when asked “If your physician prescribed a reference drug and the pharmacist offered you a more expensive generic, would you agree to switch?”, 97.1% replied that they would not. In a study conducted by Blatt et al.,^(6)^ 34.6% replied that their primary physicians never prescribe generics while only 23.5% always prescribe them. Similar findings were obtained by García el al.,^(13)^ reporting that only 22% of prescriptions had been issued under the generic nomenclature established by ANVISA.^(12)^ This fact suggests that the awareness of medical doctors should be heightened with regard to prescribing generic drugs.

Blatt et al.^(6)^ conducted a study in order to explore the level of knowledge and use of generic drugs among people living in the Tubarão municipality, Santa Catarina State. To do so, they conducted a cross-sectional study with a sample consisting of 234 subjects. In order to ascertain the knowledge of these respondents, three illustrations of two medications (paracetamol and atenolol) were presented. Each medication was shown in illustrations corresponding to the reference, generic and similar drugs. It was noted that 91.0% of the volunteers correctly identified all the figures showing generic drugs. This means that the lay population is able to recognize generic drugs and consequently, the low number of prescriptions issued by physicians, as mentioned by the respondents, cannot be blamed on possible confusion about the prescribed medication.

Along these lines, studies show that a good step for boosting generic prescriptions is to step up the level of knowledge among their prescribers.^(13, 14)^ Low prescription levels for generic drugs can be explained, as the dissemination process of reference and similar drugs is far more dynamic than that of the generics, due to the persuasive tools deployed by the pharmaceutical industry, including their sales representatives, who divulge basic information and showcase the reference drug to medical practitioners.^(22)^ This does not mean that generics are not publicized, but rather that this occurs less frequently and less intensively, compared to similar and reference drugs.^(6)^

## CONCLUSION

The studied population showed that it is endowed with sufficient knowledge about generic drugs, in terms of definitions, efficacy and cost, and consequently it can be proven that the volunteer respondents were very likely to take them. Additionally, the findings of this study also show that programs must be implemented in order to boost generic drug prescriptions among physicians.
