# Supplementary material for: Knowledge, perceptions and use of generic drugs: a cross sectional study
Source: Einstein (Sao Paulo). 2014 Jul-Sep;12(3):267–73. doi: 10.1590/S1679-45082014AO3125 (PMC4872934; doi:10.1590/S1679-45082014AO3125)
Supplement: Translation

# Translation: Conhecimento, percepções e utilização de medicamentos genéricos: um estudo transversal

**Authors:** Claudio Andre Barbosa de Lira, Jéssica Nathalia Soares Oliveira, Marília dos Santos Andrade, Cássia Regina Vancini-Campanharo, Rodrigo Luiz Vancini

**Affiliations:** 1 Setor de Fisiologia Humana e do Exercício, Faculdade de Educação Física e Dança, Universidade Federal de Goiás, Goiânia, GO, Brasil.; 2 Curso de Biomedicina, Regional Jataí, Universidade Federal de Goiás, Jataí, GO, Brasil.; 3 Departamento de Fisiologia, Universidade Federal de São Paulo, São Paulo, SP, Brasil.; 4 Escola Paulista de Enfermagem, Universidade Federal de São Paulo, São Paulo, SP, Brasil.; 5 Centro de Educação Física e Desportos, Universidade Federal do Espírito Santo, Vitória, ES, Brasil.

**Keywords:** Medicamentos de referência, Medicamentos genéricos, Políticas públicas, Uso de medicamentos, Educação do paciente

## Abstract

**Objetivo:**

Avaliar nível de conhecimento, percepções e perfil de utilização dos medicamentos genéricos entre leigos.

**Métodos:**

Realizou-se um estudo transversal com 278 voluntários (180 mulheres e 98 homens, com idade de 37,1±15,8 anos). Criou-se um questionário com perguntas em relação à utilização, à percepção e ao conhecimento sobre genéricos.

**Resultados:**

A maioria dos entrevistados (99,6%) conhecia a existência dos genéricos, e apenas 48,6% souberam definir corretamente o que os mesmos são. Do total de entrevistados, 78,8% tiveram algum tipo de informação com relação aos genéricos. Essas informações foram obtidas, principalmente, por meio da televisão (49,3%). Com relação às características dos medicamentos genéricos, 79,1% afirmaram ter confiança em sua eficácia, 74,8% acreditavam que o medicamento genérico possuía um efeito igual ao do medicamento de marca, 88,8% informaram que o genérico possuía um preço menor que o medicamento de marca, e 80,2% afirmaram comprar o medicamento genérico por conta do preço. Quanto à prescrição de medicamentos por parte dos profissionais médicos, 17,6% dos participantes afirmaram que seu médico nunca prescreveu medicamentos genéricos e apenas 7,5% disseram que seus médicos sempre prescreviam genéricos.

**Conclusão:**

Para um público leigo, a amostra estudada apresentou suficiente conhecimento com relação aos genéricos, no que concerne à definição, eficácia e custo. Dessa forma, os voluntários entrevistados apresentaram elevada propensão à utilização de genéricos. Adicionalmente, os resultados deste estudo demonstraram que se faz necessário implantar programas para aumentar a prescrição de medicamentos genéricos por profissionais médicos.

## INTRODUÇÃO

Os medicamentos desempenham papel fundamental na proteção e recuperação da saúde, além de auxiliarem na manutenção e na melhoria da qualidade de vida.^(1)^ Aproximadamente um terço da população mundial tem dificuldade de acesso a medicamentos, pelos elevados preços, sendo que essa proporção aumenta para 50% em países em desenvolvimento.^(2)^ Assim, os medicamentos genéricos são uma alternativa aos medicamentos de referência em vários países do mundo como, por exemplo, Estados Unidos, Alemanha, Reino Unido, Iraque, Malásia e Brasil.^(3)-7)^

O medicamento genérico é definido como aquele que é produzido livremente após o prazo de proteção da patente do produto de referência, devendo ser semelhante ao de referência em bioequivalência, a fim de obter o mesmo efeito terapêutico.^(8)^ O medicamento de referência é aquele registrado no órgão federal responsável pela vigilância sanitária, cuja qualidade deve ser comprovada cientificamente, por ocasião do registro, e a eficácia e a segurança devem ser testadas por meio de estudos clínicos.^(9)^ Além dos medicamentos de referência e genéricos, existe uma terceira classe denominada “medicamentos similares”, definidos como aqueles que contêm o mesmo ou os mesmos princípios ativos, mesma concentração, forma farmacêutica, via de administração, posologia e indicação terapêutica, e que é equivalente ao medicamento registrado no órgão federal responsável, podendo diferir em características relativas ao tamanho e forma do produto, prazo de validade, embalagem, rotulagem, excipientes e veículo.^(9)^

Na prática, os genéricos e similares são cópias do medicamento de referência, sendo a diferença entre eles o fato de os genéricos utilizarem o nome do princípio ativo e os similares, o nome comercial. Adicionalmente, desde sua criação, o medicamento genérico tinha como obrigatoriedade a apresentação dos testes de bioequivalência, enquanto essa obrigatoriedade, para os similares, no Brasil, foi a partir do ano de 2003. No entanto, até o fim de 2014, todos os medicamentos similares brasileiros deverão ter a comprovação da biodisponibilidade relativa.^(9)^

No Brasil, a implantação dos medicamentos genéricos (Lei 9.787 de 1999) proporcionou uma redução do preço entre 40 e 62%.^(10)^ No entanto, a venda destes em território nacional responde por apenas 27,1% do mercado farmacêutico, enquanto que nos Estados Unidos o mercado é de aproximadamente 80%.^(11)^

A despeito das importantes vantagens dos medicamentos genéricos, já foram descritos fatores de resistência com relação a seu uso como, por exemplo: (1) a baixa disponibilidade desses produtos nas farmácias;^(10)^ (2) o baixo estímulo à prescrição pelos profissionais médicos;^(6, 12)^ (3) a falta de conhecimento entre os profissionais da saúde;^(13)^ (4) a falta de orientação para o uso;^(14)^ e a (5) falta de conhecimento e as crenças negativas dos consumidores quanto à sua utilização. Vallès et al.,^(14)^ em um estudo realizado na Espanha, mostraram que 98,8% dos pacientes aceitaram trocar os medicamentos de referência por genéricos após terem recebido informações sobre os genéricos. Isso demonstra que o conhecimento a cerca dos medicamentos genéricos é um fator importante para a escolha destes pelos consumidores.

No Brasil, os gastos com assistência à saúde representam o quarto maior grupo das despesas familiares, e os gastos com medicamentos são aproximadamente 48,6% dessas despesas.^(15)^ Dessa forma, a introdução dos genéricos se tornou uma alternativa para aquisição de medicamentos de qualidade a preços acessíveis para grande parte da população.

Desse modo, um trabalho de caráter transversal, que avalie o conhecimento sobre os medicamentos genéricos, as informações recebidas pelos consumidores sobre esses medicamentos e o perfil de utilização dos consumidores, pode mostrar a situação dos mesmos no Brasil após 15 anos da promulgação da lei dos genéricos, bem como os pontos que ainda deixam dúvidas no consumidor. Além disso, esta pesquisa pode ajudar a direcionar o planejamento de políticas de saúde pública e de educação em saúde com relação a essa importante questão.

## OBJETIVO

O objetivo do presente estudo foi investigar o conhecimento, a percepções e a utilização de medicamentos genéricos entre leigos.

## MÉTODOS

### Sujeitos

Tratou-se de um estudo descritivo e transversal, do qual participaram 278 sujeitos (180 mulheres) com idade de 37,1±15,8 anos (18,1-89,7 anos, mínimo-máximo). Os voluntários foram recrutados por meio de contato direto em locais públicos, tais como vias públicas e centros comerciais, e foram oriundos dos seguintes municípios brasileiros: São Paulo (SP), com n=124; Jataí (GO), com n=110; Osasco (SP), com n=6; Guarulhos (SP), com n=5; Mairiporã (SP), com n=2; Rio Verde (GO), com n=2; Carapicuíba (SP), com n=1; Diadema (SP), com n=1; Franco da Rocha (SP), com n=1; Itaquaquecetuba (SP), n=1; Mineiros (GO), com n=1, Patos (PB), com n=1; Quirinópolis (GO), com n=1; Rio de Janeiro (RJ), com n=1; Santos (SP), com n=1, Teresina (PI), com n=1 e 19 não identificaram o município de origem. Com relação ao serviço de saúde, 158 (56,8%) entrevistados afirmaram possuir convênio médico e serem atendidos em serviços particulares; 105 (37,8%) eram atendidos em serviços públicos; 9 (3,2%) não possuíam convênio e eram atendidos em serviços públicos e particulares de saúde; e 6 (2,1%) afirmaram que não possuíam e não eram atendidos por qualquer espécie de serviço de saúde. A tabela 1 mostra as características adicionais da amostra.

**Tabela 1:** Caracterização da amostra (n=278)

Renda familiar (salário mínimo)	n (%)	Classe socioeconômica*	n (%)	Escolaridade	n (%)
Até 1	10 (3,6)	E	0 (0,0)	Ensino Fundamental incompleto	22 (7,9)
1-2	35 (12,6)	D	6 (2,2)	Ensino Fundamental completo	10 (3,6)
2-3	43 (15,5)	C2	28 (10,1)	Ensino Médio incompleto	24 (8,6)
3-4	63 (22,7)	C1	60 (21,6)	Ensino Médio completo	88 (31,7)
4-5	51 (18,3)	B2	106 (38,1)	Ensino Superior incompleto	69 (24,8)
5-10	49 (17,6)	B1	55 (19,8)	Ensino Superior completo	35 (12,6)
10-15	9 (3,2)	A2	21 (7,6)	Pós-Graduação incompleta	5 (1,8)
15-20	18 (6,5)	A1	2 (0,7)	Pós-Graduação completa	25 (9,0)

* Foi utilizado o critério da Associação Brasileira de Empresas de Pesquisa.^(16)^

Foram excluídos da amostra menores de 18 anos, estudantes ou profissionais da área da saúde, e pessoas com distúrbios cognitivos que impossibilitassem a compreensão dos instrumentos de coleta. A pesquisa foi realizada no período de março de 2012 a outubro de 2013. Todos os instrumentos de coleta e análise foram aprovados pelo Comitê de Ética em Pesquisa da Universidade Federal de Goiás (protocolo 168/2012) e seguiram a Resolução 466/2012 do Conselho Nacional de Saúde. Após a explicação detalhada dos objetivos e dos procedimentos, os indivíduos assinaram o Termo de Consentimento Livre e Esclarecido.

### Avaliação do conhecimento sobre medicamentos genéricos

Após extensa revisão da literatura sobre o assunto, foi criado um questionário (Anexo 1) para avaliar o conhecimento e a percepção sobre os medicamentos genéricos. O questionário continha três partes: (1) dados pessoais, (2) escolaridade e (3) conhecimento sobre os genéricos, experiência prévia com estes, formas de aquisição, motivos para uso e as razões que fundamentaram ou não sua escolha.

### Análise estatística

Os dados foram analisados com o programa de estatística *GraphPad Prism* ^®^ (versão 5.0, San Diego, Estados Unidos). Foi utilizada estatística descritiva para a análise dos resultados (média, desvio padrão e frequências absoluta e relativa).

## RESULTADOS

Dentre os entrevistados, 277 (99,6%) já tinham ouvido falar dos genéricos. Surpreendentemente, um voluntário respondeu que nunca tinha escutado falar sobre tais medicamentos. Adicionalmente, 135 entrevistados (48,6%) definiram corretamente o que são os medicamentos genéricos e 86 (30,9%) não souberam responder essa questão.

A despeito disso, 219 (78,8%) entrevistados tinham recebido informações em relação aos genéricos. Essas informações foram obtidas pelos seguintes meios: televisão (49,3%), profissionais médicos (18%), farmácias (por meio do balconista ou farmacêutico) (39,5%), internet e em artigos de jornais (7,2%), conhecidos ou vizinhos (3,6%), rádio (2,9%), propagandas de rua (1,8%) e outros profissionais da saúde e nas universidades (0,7%). Quanto ao uso, 225 (81%) informaram que utilizam ou já utilizaram os genéricos.

Sobre as características dos genéricos, 220 (79,1%) afirmaram ter confiança em sua eficácia, 208 (74,8%) acreditavam que estes tinham efeito igual ao dos medicamentos de referência, 209 (75,2%) acreditavam que o medicamento genérico possuía uma segurança igual ao dos medicamento de referência. Entretanto, 40 (14,4%) achavam que o genérico possui uma qualidade inferior ao do medicamento de referência e 95 (34,2%) não escolheriam o genérico em detrimento do medicamento de referência.

Quanto à prescrição de medicamentos por parte dos médicos, 49 (17,6%) participantes afirmaram que seus médicos nunca prescreveram medicamentos genéricos e apenas 21 (7,5%) disseram que seus médicos sempre prescreviam genéricos. Apesar da baixa prescrição por parte dos profissionais médicos, 48 voluntários (17,4%) afirmaram que sempre costumavam comprar esses medicamentos, 62 (22,3%) frequentemente compravam e 107 (38,5%) compravam genéricos às vezes.

Entre os entrevistados, 247 (88,8%) informaram que o genérico possuía um preço menor que o medicamento de referência e 223 (80,2%) afirmaram comprar o genérico por conta do preço.

Quando questionados “Se o seu médico prescreveu um medicamento de marca e o farmacêutico lhe oferece um medicamento genérico mais barato, você aceita a troca?”, 183 (65,8%) responderam que sim. Quando questionados “Se o seu médico prescreveu um medicamento de marca e o farmacêutico lhe oferece um medicamento genérico de mesmo preço, você aceita a troca?”, apenas 74 (26,6%) responderam que sim. Quanto à afirmativa “Somente troco o medicamento de referência pelo medicamento genérico, se a doença não for grave”, 93 (33,5%) responderam verdadeiro.

Dos entrevistados, 157 sujeitos (56,5%) afirmaram que os genéricos possuíam a mesma substância que o de referência e 140 (50,3%) consideravam boa a divulgação dos genéricos no Brasil. Quando interrogados se encontravam medicamentos genéricos nas farmácias, 120 (43,2%) afirmaram que sempre os encontravam.

## DISCUSSÃO

No Brasil, os gastos com medicamentos representam 48,6% das despesas com saúde.^(15)^ Desse modo, a disponibilidade de medicamentos a preços acessíveis deve constar na pauta das políticas de saúde pública. Alguns estudos avaliaram a adesão à utilização dos genéricos no Brasil^(16, 17)^ e, no início, por desconhecimento entre os profissionais da saúde e pela população leiga, existia muita resistência à prescrição e ao seu uso. Com o objetivo de verificar se esse quadro mudou, o presente estudo investigou as percepções, o nível de conhecimento e a utilização dos genéricos após 15 anos da promulgação da lei dos medicamentos genéricos no Brasil e evidenciou-se que 99,6% dos entrevistados já tinham ouvido falar deles. Esse resultado é semelhante ao obtido em outros estudos realizados no Brasil.^(11, 17)^ Em uma pesquisa feita no Recife (PE), da Rocha et al.^(17)^ demonstraram que 95,7% dos entrevistados tinham ouvido falar dos genéricos. Em uma pesquisa realizada pela Agência Nacional de Vigilância Sanitária (ANVISA), 95% dos consumidores afirmaram conhecer os genéricos.^(11)^ Felizmente, o cenário brasileiro é melhor que o observado em outros países. Em um estudo feito na cidade de Auckland, na Nova Zelândia, apenas 51% dos entrevistados tinham ouvido falar do termo “medicamento genérico”.^(3)^ Naing et al.^(18)^ avaliaram o conhecimento de medicamentos utilizados pela população da Malásia e verificaram que 85,8% não conheciam o termo “medicamento genérico”, 86,3% não sabiam responder com relação à qualidade dos genéricos quando comparados ao medicamento de referência e 86,9% não sabiam a diferença no preço entre o genérico e o medicamento de referência. Pode-se atribuir o bom resultado obtido na população brasileira aos programas de conscientização e de popularização dos medicamentos genéricos fomentados pelo governo brasileiro e pelos laboratórios produtores desse tipo medicamentos.

No presente estudo, 78,8% obtiveram informações sobre os genéricos e, destes, 49,3% obtiveram informações por meio da televisão e 18% por profissionais médicos. Resultados semelhantes aos do presente estudo foram descritos por Himmel et al.,^(19)^ em um estudo realizado na população alemã.

Com relação à definição de medicamento genérico, 48,6% dos entrevistados o definiram corretamente. Em um estudo conduzido na Malásia com 216 pessoas, Thomas e Vitry^(7)^ avaliaram o conhecimento sobre os genéricos, o desejo de usá-los e as razões da escolha. Da amostra avaliada, 32,5% afirmaram que conheciam o que eram os medicamentos genéricos. Entretanto, quando solicitados a fornecer uma definição mais exata, 7% (dos 32,5%) foram incapazes de fazê-lo. Daqueles que a forneceram, 51% disseram que eram medicamentos mais baratos, 18% afirmaram que eram medicamentos “não originais” ou “não genuínos”, 18% afirmaram que eram medicamentos feitos localmente ou por uma companhia diferente da tradicional, e 13% afirmaram ser uma marca diferente da de um medicamento com o mesmo conteúdo.

No presente estudo, a maioria dos entrevistados (79,1%) acreditava na eficácia dos genéricos. Resultados semelhantes ao nosso estudo foram obtidos por Thomas e Vitry.^(7)^ Provavelmente, aquelas pessoas que referiram não acreditar na eficácia dos medicamentos genéricos o fizeram por terem tido uma experiência negativa com tais medicamentos (pior eficácia quando comparados com os medicamentos de referência). Entretanto, tal efeito não é uma exclusividade dos genéricos. Pode-se atribuir isso à variação interindividual, que é um sério problema em que pode existir perda de eficácia farmacológica e efeitos adversos. Alguns fatores, que estão associados com a variabilidade dos efeitos farmacológicos e terapêuticos, são a idade, a gravidez e presença de doença.^(20)^ De fato, existem situações nas quais determinado medicamento pode apresentar resposta terapêutica diferente do esperado ou, ainda, em alguns casos, o paciente é refratário ao tratamento farmacológico como, por exemplo, um terço dos pacientes com depressão são refratários ao tratamento farmacológico.^(21)^ Apesar de alguns terem resistência à utilização dos genéricos, no presente estudo, 80,9% dos entrevistados já tinham utilizado essa terapia. Tal resultado não pode ser atribuído à prescrição destes pelos profissionais médicos, pois, de acordo com os entrevistados, somente 7,5% e 13,3% sempre e frequentemente, respectivamente, prescreviam os genéricos. Este resultado é digno de nota, pois sugere que a população não respeita a prescrição médica e substitui o medicamento de referência pelo genérico mais barato. No presente estudo, 88,8% assinalaram que o genérico é mais barato que o medicamento de referência, 80,2% compravam o medicamento genérico por conta do preço e 65,8% dos entrevistados responderam afirmativamente quando perguntados se escolheriam o genérico em detrimento do medicamento de referência. Finalmente, quando perguntados “Se o seu médico prescreveu um medicamento de marca e o farmacêutico lhe oferece um medicamento genérico mais barato, você aceita a troca?”, 65,8% responderam afirmativamente. Isso denota que o preço do medicamento genérico é um fator decisivo para a escolha feita pelo paciente. Corroborando essa afirmação, quando os entrevistados foram perguntados “Se o seu médico prescreveu um medicamento de marca e o farmacêutico lhe oferece um medicamento genérico de mesmo preço, você aceita a troca?”, 73,4% responderam negativamente e quando foram perguntados “Se o seu médico prescreveu um medicamento de referência e o farmacêutico lhe oferece um genérico mais caro, você aceita a troca?”, 97,1% responderam negativamente. Em um estudo conduzido por Blatt et al.,^(6)^ 34,6% responderam que os profissionais médicos que costumavam consultar nunca prescreviam os genéricos e apenas 23,5% sempre prescreviam. Resultados semelhantes foram obtidos por García el al.,^(13)^ que relataram que apenas 22% das prescrições haviam sido feitas pela nomenclatura genérica da ANVISA.^(12)^ Tal fato sugere que os profissionais médicos deveriam ser sensibilizados com relação à prescrição dos genéricos.

Blatt et al.^(6)^ conduziram um estudo com o objetivo de pesquisar o nível de conhecimento e o grau de utilização de genéricos em residentes do município de Tubarão (SC). Para tanto, realizaram um estudo transversal com uma amostra constituída por 234 sujeitos. Para verificar o conhecimento dos entrevistados, foram apresentadas três ilustrações de dois medicamentos (paracetamol e atenolol). Cada medicamento possuía uma ilustração, que correspondia ao medicamento de referência, genérico e similar. Observou-se que 91,0% dos voluntários identificaram corretamente todas as figuras de medicamentos genéricos. Isso denota que a população leiga sabe reconhecer um medicamento genérico e que, portanto, o baixo número de prescrições, pelos médicos, apontado pelos entrevistados, não pode ser atribuído a uma possível confusão do medicamento prescrito.

Nesse sentido, estudos demonstram que uma boa medida para aumentar a prescrição de genéricos é ampliar o nível de conhecimento de quem os prescreve.^(13, 14)^ A baixa prescrição dos genéricos pode ser explicada, pois os medicamentos de referência e similares têm seu processo de difusão muito mais dinâmico que os genéricos, devido aos instrumentos de persuasão da indústria farmacêutica, entre os quais figuram os representantes farmacêuticos, que divulgam as informações básicas e fazem propaganda do medicamento de referência entre os profissionais médicos.^(22)^ Isso não significa que os genéricos não sejam propagandeados, mas sim que isso ocorre com menores frequência e intensidade em relação aos medicamentos similares e de referência.^(6)^

## CONCLUSÃO

A população estudada demonstrou que possuía conhecimento suficiente em relação aos genéricos, no que concerne à definição, à eficácia e ao custo, e consequentemente, é provável que os voluntários entrevistados apresentem elevada propensão à utilização dos mesmos. Adicionalmente, os resultados do presente estudo ainda demonstram que se faz necessária a implantação de programas com o intuito de aumentar a prescrição de medicamentos genéricos pelos profissionais médicos.
